# Extravascular Lung Water Does Not Increase in Hypovolemic Patients after a Fluid-Loading Protocol Guided by the Stroke Volume Variation

**DOI:** 10.1155/2012/437659

**Published:** 2012-10-04

**Authors:** Carlos Ferrando, Gerardo Aguilar, F. Javier Belda

**Affiliations:** ^1^Department of Anesthesiology and Critical Care, Hospital Clínico Universitario de Valencia, 46010 Valencia, Spain; ^2^Department of Surgery, School of Medicine, University of Valencia, 46010 Valencia, Spain

## Abstract

*Introduction*. Circulatory failure secondary to hypovolemia is a common situation in critical care patients. Volume replacement is the first option for the treatment of hypovolemia. A possible complication of volume loading is pulmonary edema, quantified at the bedside by the measurement of extravascular lung water index (ELWI). ELWI predicts progression to acute lung injury (ALI) in patients with risk factors for developing it. The aim of this study was to assess whether fluid loading guided by the stroke volume variation (SVV), in patients presumed to be hypovolemic, increased ELWI or not. *Methods*. Prospective study of 17 consecutive postoperative, fully mechanically ventilated patients diagnosed with circulatory failure secondary to presumed hypovolemia were included. Cardiac index (CI), ELWI, SVV, and global end-diastolic volume index (GEDI) were determined using the transpulmonary thermodilution technique during the first 12 hours after fluid loading. Volume replacement was done with a strict hemodynamic protocol. *Results*. Fluid loading produced a significant increase in CI and a decrease in SVV. ELWI did not increase. No correlation was found between the amount of fluids administered and the change in ELWI. *Conclusion*. Fluid loading guided by SVV in hypovolemic and fully mechanically ventilated patients in sinus rhythm does not increase ELWI.

## 1. Introduction

Circulatory failure secondary to hypovolemia is a common situation in critical care patients. Volume replacement is the first option for the treatment of hypovolemia [1]. Adequate fluid replacement improves stroke volume (SV) according to the Frank-Starling relationship. An increase in ventricular preload will increase SV and therefore cardiac index (CI) which in turn will improve oxygen delivery. Fluid management is an essential part of the treatment of hypovolemic critically ill patients, but inappropriate fluid loading increases the risk of developing pulmonary edema [1, 2]. 

Recently, a number of papers have been published assessing which parameters are most accurate for predicting fluid responsiveness. Many papers have shown that in patients in sinus rhythm during controlled mechanical ventilation the dynamic parameters (SVV; pulse pressure variation: PPV) predict more accurately fluid responsiveness than the static parameters (intrathoracic blood volume index: ITBI; central venous pressure: CVP) [3–15]. These papers used different protocols for fluid loading which also differed in the quantity, the type of fluid, and even the duration of the administration.

Many papers note that inadequate fluid loading increases the risk of developing pulmonary edema, but few papers actually determine or quantify pulmonary edema after volume replacement. Additionally, to date, no study has been performed to determine if fluid loading guided by dynamic parameters (SVV) increases pulmonary edema using the measurement of extravascular lung water, as has been recommended in previous studies [16, 17]. On the other hand, ELWI has been shown to be a good predictor of mortality in critically ill patients [18]. 

The aim of our study was to demonstrate whether fluid loading guided by SVV in patients presumed to be hypovolemic and fully mechanically ventilated increases extravascular lung water or not. 

## 2. Materials and Methods

This was a prospective study that including 17 consecutive patients (from March to September 2010) with circulatory failure secondary to presumed hypovolemia during the first 24 hours of the postoperative period. Written informed consent was obtained from relatives. The study was approved by the Ethical Committee of the Hospital Clínico Universitario of Valencia. Inclusion criteria were patients in sinus rhythm on controlled ventilation with signs of circulatory failure secondary to presumed hypovolemia. Presumed hypovolemia [16] was arbitrarily defined as hypotension (systolic arterial pressure—SP—below 90 mmHg) or one of the following; the need for inotropes or vasopressors to maintain SP over 90 mmHg; a decrease in SP of more than 40 mmHg; oliguria; blood lactate above 2 mmol/L; cardiac index (CI) below 3.5 L/min/m^2^; GEDI below 680 mL/m^2^; SVV above 10%. Exclusion criteria were cardiac arrhythmias, aortic valvular stenosis or regurgitation, known intracardiac shunt, extravascular lung water index (ELWI) above 12 mL/kg, and Pulmonary Vascular Permeability Index (PVPI) above 3 or a hematocrit lower than 28%. The severity of illness of the patients was determined according to the Sequential Organ Failure Assessment (SOFA) score.

As SVV can only be used as a marker of fluid responsiveness in patients who are fully mechanically ventilated [19], prior to inclusion in the study patients were sedated and mechanically ventilated in a volume-controlled mode (VCM) using the following parameters: tidal volume (VT) of 8 mL/kg, positive end-expiratory pressure (PEEP) between 5 and 10 cmH_2_O, plateau pressure (Ppt) below 30 cmH_2_O, and a respiratory rate (RR) to maintain a PaCO_2_ between 35 and 45 mmHg. 

**Figure 1:**
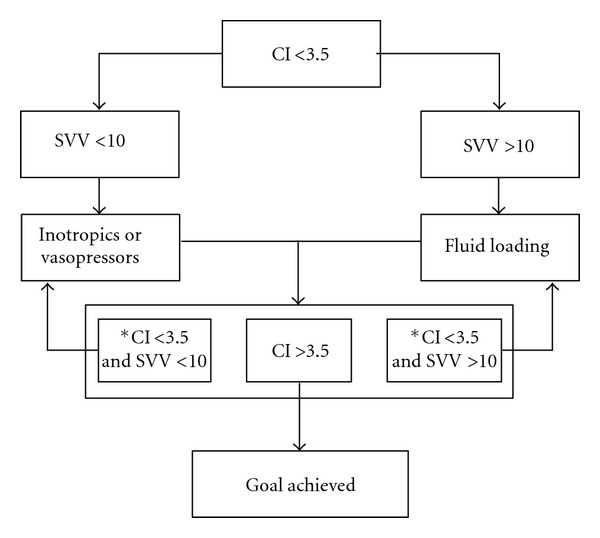
Treatment algorithm.  CI cardiac index (L/min/m^2^); SVV: stroke volume variation (%). *If CI < 3.5 L/min/m^2^ and SVV < 10% with mean arterial pressure > 70 mmHg and cardiac function index (CFI) > 4 min^−1^, the patient was excluded from the study (see text). When the hematocrit was below 28%, red blood cells were transfused until its value was above 28%.

The hemodynamic variables were recorded via an arterial 5F thermistor tipped catheter, (Pulsiocath PV2015, Pulsion Medical Systems; Munich, Germany) placed in the femoral artery and connected to the PiCCO monitor (Pulsion Medical Systems, Munich, Germany). CI, GEDI, ELWI, and PVPI were determined by transpulmonary thermodilution. This method has been described previously in many papers [16–18]. Measurements of transpulmonary haemodynamic variables involved venous injection of 15 mls of ice-cold saline. The average of triplicate measurements are given. The systemic vascular resistance index (SVRI), mean arterial pressure (MAP), SVV, and stroke volume index (SVI) were recorded by the continuous pulse contour analysis method. The cardiac function index (CFI) was calculated using the following formula: CFI: CI/GEDI. The variables PaO_2_/FiO_2_, PaCO_2_, and blood lactate were obtained with an arterial blood gasometry, from blood taken from the femoral artery catheter, with a gas analyzer (RadiometerTM, Copenhagen, Denmark). Once the patient was included in the study and monitored with the transpulmonary thermodilution technique, treatment was started according to the volume replacement algorithm (Figure 1). The target for the volume replacement was to obtain a CI greater than or equal to 3.5 L/min/m^2^.

### 2.1. Treatment Algorithm (see Figure 1)

If CI was below 3.5 L/min/m^2^ and SVV above 10%, 250 mL of 6% hydroxyethyl starch 130/0.4 (Voluven; Fresenius Kabi, Bad Homburg, Germany) was administered over 15 minutes. If there was an increase in the CI above 10% and the SVV was still greater than 10%, then another 250 mL of Voluven was administered in 15 min and so on if there was an increase of CI > 10% and SVV > 10% and GEDI ≤ 850 mL/m^2^.

If the CI was below 3.5 L/min/m^2^ but the SVV was also below 10% or the increase in CI after volume replacement was below 10%, an inotrope (dobutamine, maximum dose: 10 mcg/kg/min) was administered until the target was achieved (CI > 3.5 L/min/m^2^). After this, if CI was still below 3.5 L/min/m^2^ and/or the SVV was below 10% the patient was excluded from the study and an echocardiography was performed, suspecting right heart failure or pulmonary hypertension.

Data recording was done before and 10 min after each fluid loading. After the last fluid loading, data collection was continued at hours 2, 6, and 12. All data were indexed to the predicted body weight (PBW) where appropriate. 

### 2.2. Statistics

Data was analyzed by the SPSS statistics software (version 15.0, SPSS Inc., Chicago, IL). To determine a difference in ELWI of 20%, considered as clinically significant, with a power of 80%, and a significance of 95% (*P* < 0.05), a sample size of 17 was calculated. The data was given as mean ± standard deviation (SD). To determine if significant differences existed after fluid loading, the information was analyzed by exact ANOVA test of repeated measurements by the Bonferroni post hoc test. To determine the relation between the SVV and the GEDI determined prior to fluid replacement (T0) and the change in CI responsiveness, the Pearson correlation coefficient was used. The interrelation between changes (Δ) in CI, SVV, and GEDI as well as between the administered volume and the ELWI also were analysed using the Pearson's correlation after normal distribution of all the parameters.

## 3. Results

Seventeen patients admitted to our Surgical Intensive Care Unit (SICU) were included in the study with twenty five patients screened in total (8 patients had exclusion criteria: three with an ELWI > 12 mL/kg and five with atrial fibrillation). The demographic information and the respiratory parameters of the patients, diagnosis, and SOFA scores are shown in Table 1. Ten (58%) of the patients included in the study needed vasopressor support (norepinephrine 0.05–0.1 mcg/kg/min) from the beginning of the study. After volume replacement, vasopressor use was ceased in seven of these patients, and in three of them doses were reduced. The mean fluid balance during the study protocol was +395 mL (with a standard deviation of ±70 mL). As can be seen in Table 2, after fluid loading CI increased significantly in the first 6 hours (*P* < 0.001). SVV decreased significantly after fluid loading for the next 12 hours (*P* < 0.001). There was a nonsignificant increase in the volumetric dynamic parameter, stroke volume index SVI (*P* = 0.168) and the static parameters GEDI (*P* = 0.456) and CVP (*P* = 0.151) and the contractility parameter CFI (*P* = 0.706). After volume replacement there were no changes in ELWI (*P* = 0.993) or PVPI (*P* = 0.748) for the duration of the study (12 hours). There was no significant difference in the Plateau pressure (Ppt) (*P* = 0.489) (Table 1). 

**Figure 2:**
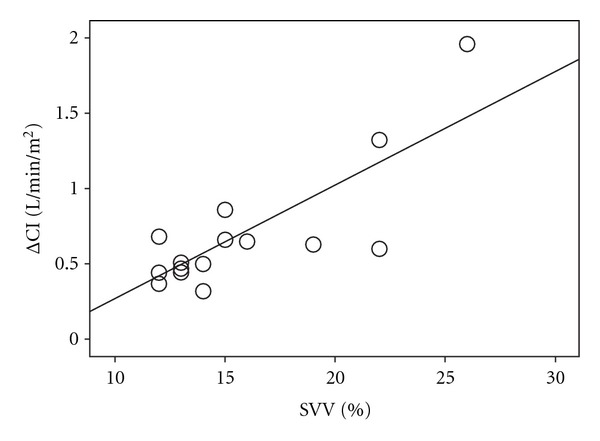
Pearson's correlation between the variations of cardiac index (ΔCI, L/min/m^2^) and the baseline values of stroke volume (SVV, %) after fluid loading (*r* = 0.81, *P* < 0.001).

**Figure 3:**
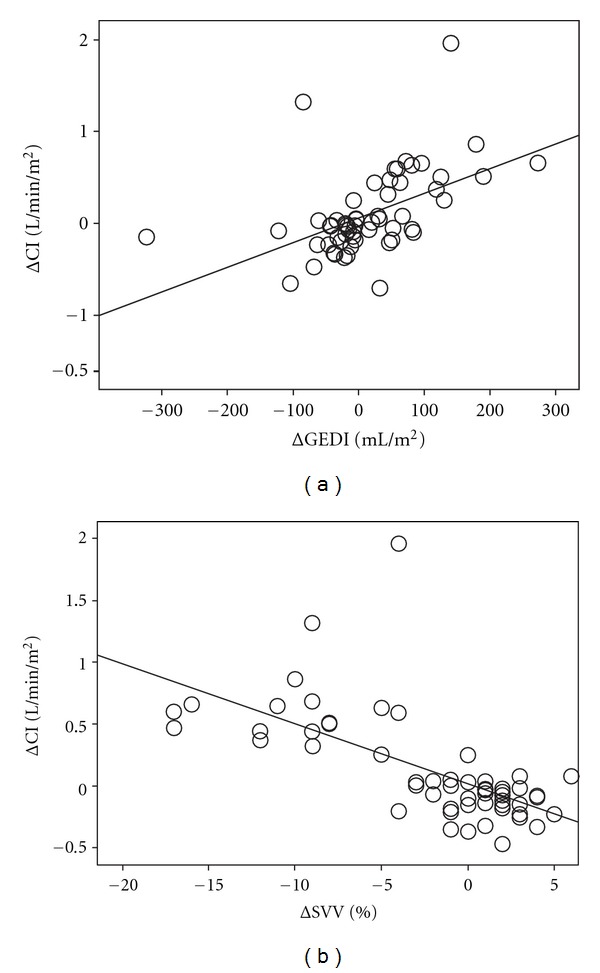
Pearson's correlation between (a) the variations of cardiac index (ΔCI, L/min/m^2^) and the global end diastolic index (ΔGEDI, mL/m^2^) (*r* = 0.51, *P* < 0.001) and (b) the variations of cardiac index (ΔCI, L/min/m^2^) and the stroke volume variation (ΔSVV, %) (*r* = 0.68, *P* < 0.0001).

No correlations were found between the amount of fluids administered and the change in ELWI (T1, *P* = 0.945, *r* = 0.019; T2, *P* = 0.867, *r* = 0.047; T6, *P* = 0.697, *r* = 0.110; T12, *P* = 0.826, and *r* = 0.68).  Figure 2 shows a significant correlation between baseline values of the SVV (*P* < 0.001, *r* = 0.802) and the changes in CI. This correlation was not found for the GEDI (*P* = 0.561, *r* = − 0.163) or the CVP (*P* = 0.585, *r* = −0.154). After fluid loading, the changes in the CI (ΔCI) significantly correlated with the changes to the SVV (ΔSVV) (*P* < 0.001, *r* = − 0.684) and the changes in GEDI (ΔGEDI) (*P* < 0.001, *r* = 0.511) (Figure 3). The information obtained by the blood gas analysis (Table 3) also highlights the tendency towards decrease of the blood lactate during resuscitation and the slight improvement in oxygenation (PaO_2_/FiO_2_).

## 4. Discussion

Circulatory failure secondary to hypovolemia is a common situation in critical care patients. Volume replacement is the first option for the treatment of hypovolemia [1]. Prediction of fluid responsiveness is part of the basic management of critical care patients. Inappropriate fluid loading increases the risk of developing pulmonary edema [1, 2]. Additionally, pulmonary edema increases the risk of acute lung injury [17, 20] and therefore mortality [17, 18] in the critical care patients. 

We have demonstrated in postoperative patients that individualized fluid therapy guided by a dynamic parameter (SVV), increases CI but does not increase pulmonary edema, as quantified by extravascular lung water. It is possible that the same results were obtained if other dynamic parameters like pulse pressure variation (PPV) or systolic pressure variation (SVP) were used [3–15]. 

Another sign that fluid loading did not increase pulmonary edema was that the oxygenation (PaO_2_/FiO_2_ ratio) was maintained for the duration of the study (Table 3). This suggests the absence of alveolar collapse. Previous studies suggest that an increase in EVLW produces a decrease in the PaO_2_/FiO_2_ ratio as a consequence of the ventilation perfusion mismatch associated with pulmonary edema [21]. These results are very relevant because adequate fluid therapy decreases the risk of developing acute lung injury (ALI) [20] and therefore mortality [18]. Also, our population was at an increased risk for developing pulmonary edema. Most of our patients already had high PVPI values at the beginning of the study. An increase in ELWI after fluid loading as a consequence of alterations of the pulmonary vascular permeability has been shown to reflect the changes usually induced in patients following cardiac surgery [22], sepsis [23], or traumatic brain injury [24]. In our group of patients ELWI and PVPI did not change after fluid loading (Table 2). 

Similar results have been found in previous studies but, in contrast to our study where fluid loading was guided by a dynamic parameter (SVV), all of these studies used static parameters to guide their fluid management. Matejovic et al. [25] concluded that with individualized fluid loading guided by central venous pressure (CVP) and pulmonary artery occlusion pressure (PAOP), patients will never reach the steep part of the cardiac function curve. This produces an efficient expansion of plasma without intravascular overload which is favored by mechanical ventilation with PEEP that limits the accumulation of ELWI. Verheij et al. [26] studied fluid loading with four different types of fluids in the postoperative period of cardiac surgery. Independent of the type of fluid used, if fluid loading was guided by CVP or PAOP, ELWI did not increase. Bindels et al. [27] performed a study with a methodology similar to ours in septic patients. They guided fluid loading (500 mL colloid) with static parameters like CVP, PAOP, or intrathoracic blood index (ITBI). During the first 24 hours after fluid loading ELWI did not increase. 

Even though the results from these studies are very important, other studies have demonstrated that static pressure and volumetric indices are of poor predictive value whereas dynamic parameters have been proven to be both predictive and reliable [3–15]. 

In our study SVV was the only hemodynamic parameter able to predict fluid responsiveness. When fluid loading increased the CI values to over 3.5 L/min/m^2^ there was a simultaneous and significant reduction in SVV. But the static parameters such as the GEDI or the CVP only changed in a nonsignificant way. Also, the static parameter GEDI correlated with the CI after fluid loading but was not able to predict fluid responsiveness. 

These results confirm those obtained by Reuter et al. [28] in postoperative cardiac surgery and Berkenstadt et al. [29] in postoperative brain surgery. They showed that the dynamic parameter (SVV) was better for predicting and monitoring fluid responsiveness than static parameters (CVP and PAOP). 

Our study also showed that independent of the amount of fluids administered, fluid loading did not increase ELWI in normovolemic or hypovolemic patients (GEDI < 850 mL/m^2^) with adequate fluid responsiveness, as indicated by a SVV greater than 10%. As we observed in our results, there was no correlation between the volume of fluids administered and the ELWI, even though most patients in the study had baseline values of GEDI greater than 680 mL/m^2^, although the CI was below 3.5 L/min/m^2^ and the SVV greater than 10%.

These results are similar to those obtained by Holm et al. [30] in normovolemic and hypovolemic burn patients (ITBI < 1000 mL/m^2^). They observed that there was no relationship between the volume of crystalloid administered and the ELWI. 

Our study has some limitations. First, it is important to emphasise that SVV is only valid in patients in sinus rhythm with fully controlled mechanical ventilation and we cannot extrapolate the results to other patients who do not fulfil the inclusion criteria for the study. Second, we cannot exclude the fact that ELWI may increase if fluid loading is done by using another type of fluid (such as crystalloids) or with greater volume (more than 1 liter). Third, the use of vasopressors in some of our patients could confuse our data. Several studies have demonstrated the effects of endogenous and exogenous catecholamines on unstressed-blood volume showing that these drugs can decrease SVV [31–34]. Fourth, an inclusion of a control group (without protocol-guided fluid loading or static parameters-guided) would be, from the methodological point of view, more appropriate to demonstrate clinical advantage of the tested approach. Finally the protocol only lasted for 12 hours, therefore we cannot exclude any increases in the ELWI after this time. However if should also be born in mind that ELWI may increase from other causes during this period. 

## 5. Conclusions

Fluid loading guided by stroke volume variation in hypovolemic and fully mechanically ventilated patients in sinus rhythm does not increase ELWI. Consequently, individualized volume loading guided by systolic volume variation appears to decrease the risk of developing acute lung injury in this type of patients. However, further studies are needed to evaluate if the use of greater amount of fluids does not increase the ELWI when this approach is applied.

## Figures and Tables

**Table 1:** Patients characteristics.

Patient	Age	PBW	Diagnostic	SOFA	VT	PEEP	Ppt 0	Ppt 1	FL
1	66	70,4	CABG	5	520	5	14	15	500
2	34	70,4	Brain injury	8	600	5	17	16	1000
3	74	56,8	CABG	10	480	12	15	17	750
4	55	47,2	Abdominal surgery	11	450	12	25	21	500
5	58	75	Abdominal surgery	9	530	12	22	20	750
6	82	70,4	Abdominal surgery	7	480	5	16	14	750
7	78	70,4	CABG	9	500	5	23	21	250
8	77	79,5	CABG	3	500	5	17	17	500
9	69	72,2	Thoracic surgery	6	540	5	19	18	500
10	58	56,8	CABG + MVP	8	520	9	20	21	500
11	76	75	Abdominal surgery	9	500	10	18	16	750
12	28	55	Brain injury	8	440	7	16	15	250
13	81	47,2	CABG	3	450	6	16	16	250
14	63	70,4	CABG + MVP	5	520	5	16	16	250
15	75	73,2	Abdominal surgery	11	500	10	19	18	500
16	72	55,4	Abdominal surgery	8	460	8	20	21	750
17	69	55	Abdominal surgery	9	450	6	17	17	1000

Total	63 ± 15	63 ± 10		7 ± 2	494 ± 41	7 ± 2	18 ± 3	17 ± 2	519 ± 246

Data from each patient are shown as absolute values. Data of all patients (Total) are shown as mean ± SD. PBW (Kg): predicted body weight. Diagnostics: CABG: coronary artery bypass grafting; MVP: mitral valve replacement. SOFA score: *Sequential Organ Failure Assessment* score. VT: tidal volume (mL); PEEP: positive end-expiratory pressure (cmH_2_O); Ppt 0 and Ppt 1: plateau pressure at 0 and 12 hours, respectively, (cmH_2_O). FL: fluid loading (mL).

**Table 2:** Hemodynamic variables for each time interval.

	T0	T1 (10 min)	T2 (2 h)	T3 (6 h)	T4 (12 h)
CI	2,5 ± 0,5	3,6 ± 0,6*	3,5 ± 0,6*	3,5 ± 0,6*	3,3 ± 0,9
SVV	19 ± 4	9 ± 4*	9 ± 4*	9 ± 4*	13 ± 6*
ELWI	8 ± 2	8 ± 2	8 ± 2	9 ± 1	8 ± 2
PVPI	2,1 ± 0,6	1,9 ± 0,5	1,9 ± 0,4	1,8 ± 0,4	1,9 ± 0,5
GEDI	681 ± 143	776 ± 154	737 ± 126	737 ± 137	716 ± 129
CVP	10 ± 2	13 ± 4	12 ± 4	11 ± 4	11 ± 2
MAP	74 ± 10	82 ± 8	79 ± 8	77 ± 9	77 ± 5
HR	87 ± 20	87 ± 18	87 ± 16	83 ± 17	88 ± 14
SVI	30 ± 8	39 ± 11	35 ± 9	35 ± 9	33 ± 7
SVRI	2074 ± 597	1529 ± 359*	1743 ± 310	1793 ± 453	1808 ± 540
CFI	3,7 ± 0,9	4,0 ± 1,0	4,1 ± 0,9	4,1 ± 1,07	3,9 ± 0,8

Data are shown as mean ± SD. *Statistical significance (*P* < 0,05) when the value was compared with T0 (baseline values). ELWI: extravascular lung water index (mL/kg); CI: cardiac index (L/min/m^2^); SVV: stroke volume variation (%); SVI: stroke volume index (mL/m^2^); PVPI: pulmonary vascular permeability index; SVRI: systemic vascular resistance index (dynes/sec/cm^5^/m^2^); GEDI: global end-diastolic index (mL/m^2^); CFI: cardiac function index (L/min); MAP: mean arterial pressure (mmHg); HR: heart rate (beats/min); CVP: central venous pressure (mmHg).

**Table 3:** Arterial blood gases parameters for each time interval.

	T0	T1 (10 min)	T2 (2 h)	T3 (6 h)	T4 (12 h)
PaO_2_/FiO_2_	262 ± 76	271 ± 62	285 ± 48	276 ± 47	292 ± 50
PaCO_2_	40 ± 3	41 ± 4	40 ± 4	41 ± 3	40 ± 5
Lact^−1^	1,9 ± 1,0	1,8 ± 1,0	1,7 ± 0,9	1,6 ± 0,7	1,5 ± 0,6
ABE_c_	−3,6 ± 2,1	−3,4 ± 2,0	−2,9 ± 2,2	−2,2 ± 1,9	−2,3 ± 2,0

Data are shown as mean ± SD. Statistical significance (*P* < 0.05) when the value was compared with T0 (baseline values). PaO_2_/FiO_2_: arterial oxygen tension to inspired oxygen fraction ratio (mmHg); PaCO_2_, carbon dioxide partial pressure (mmHg); Lact^−1^: Blood Lactate (mmol/L). ABE_c_: deficit base (mmol/L).
